# Preparedness of medical students to provide nutrition care following a nutrition education intervention

**DOI:** 10.1186/s13104-023-06348-5

**Published:** 2023-05-23

**Authors:** Bright Yammaha Amoore, Patience Kanyiri Gaa, Shamsu-Deen Ziblim, Victor Mogre

**Affiliations:** 1 Department of Health Professions Education and Innovative Learning, School of Medicine, University for Development Studies, P. O. Box TL 1883, Tamale, Ghana; 2 Department of Dietetics, School of Allied Health Sciences, University for Development Studies, Tamale, Ghana; 3 Department of Population and Reproductive Health, School of Public Health, University for Development Studies, Tamale, Ghana

**Keywords:** Preparedness, Nutrition education, Medical students, Relevance, Ghana

## Abstract

**Background:**

Globally, 71% of deaths are due to non-communicable diseases (NCDs) of which 77% of these deaths occur in low-and-middle income countries. Nutrition is an important contributor to the occurrence, progression and management NCDs. Health care professionals’ promotion of the adoption of healthy dietary habits among individuals has been shown to reduce the occurrence of NCDs. We assessed the effects of a nutrition education intervention on medical students’ self-perceived preparedness to provide nutrition care.

**Methods:**

We administered a pre, post and four-weeks follow-up questionnaire to second year medical students who participated in a nutrition education intervention that adapted varied teaching and learning activities. Outcomes were self-perceived preparedness, relevance of nutrition education and perceived need for further training in nutrition. Repeated measures and Friedman tests were used to assess differences in mean scores across pre, post and 4-weeks follow up assessment based on p < 0.5 at 95% confidence interval.

**Results:**

The proportion of participants who felt prepared to provide nutrition care increased significantly (p = 0.001) from 38% (n = 35) at baseline to 65.2% (n = 60) immediately post-intervention and to 63.2% (n = 54) at 4-weeks follow-up. At baseline, 74.2% (n = 69) of the students perceived nutrition education to be relevant to their future career as medical doctors which increased to 85% (n = 78) immediately after the intervention (p = 0.026) and to 76% (n = 70) 4-weeks follow-up. The proportion of participants who reportedly said they will benefit from further training in nutrition increased from 63.8% (n = 58) at pre-intervention to 74.0% (n = 68) at post-intervention (p = 0.016).

**Conclusion:**

An innovative, multiple-strategy nutrition education intervention can improve medical students’ self-perceived preparedness to provide nutrition care.

**Supplementary Information:**

The online version contains supplementary material available at 10.1186/s13104-023-06348-5.

## Background

Globally, 41 million people die each year due to non-communicable diseases (NCDs) in which 77% occur in low-and middle-income countries [1]. About 43% of these deaths are due to cardiovascular diseases, 9.3 million due to cancers, 4.1 million due to respiratory illness and 1.5 million due to diabetes [1]. Nutrition is a critical denominator of three out of the four NCDs accounting for the 71% of deaths globally. The consumption of healthy meals has been linked to a reduction in the occurrence of these chronic diseases.

Doctors play a critical role in helping individuals to adopt healthy dietary habits. Studies from the US and other developed countries have shown that patients consider doctors as credible sources of nutrition information and care [2–4]. Doctors drive the care process and decides and provides the kind of care a patient should receive. They are opportune to advocate for and refer patients to receive specialist dietetic and nutrition care from qualified healthcare professionals [5]. As such patients are more likely to adhere to dietary recommendations either from the doctor or a dietician/nutritionist if the doctor recommends it.

However, most doctors do not frequently offer nutrition care to their patients [6–9] due to a number of factors including poor nutrition-related knowledge, poor confidence to provide nutrition care, lack of time, and among others [8–16]. This situation probably sterns from previous reports that both medical students and doctors reportedly received inadequate nutrition education during training in medical school due to a number of factors including an already overcrowded medical curriculum, inadequate time allocation for nutrition resulting in them reportedly feeling inadequately trained and unprepared to provide nutrition care in the general practice setting [17–20].

In order to respond to these needs towards improving nutrition education in the medical curriculum, we designed an intervention to provide medical students with additional nutrition education. The current study intends to investigate the effect of the intervention on medical students’ self-perceived preparedness to provide nutrition care, perception of the relevance of nutrition education to their future career and their perceived need for further training in nutrition education. We also report medical students’ assessment of the quality of the intervention, implementation challenges and suggestions on how the intervention can be fine-tuned to meet their nutrition education needs.

## Methods

### Study design and setting

 A pre-post quasi-experimental design without control group (CG) was adopted for the study. which was conducted at the University for Development Studies School of Medicine (UDS-SoM), that runs a problem-based learning, community-based education and service (PBL-COBES) curriculum for undergraduate medical education. As described elsewhere [21–23], the PBL/COBES curriculum is executed through system-based course modules or blocks in which teaching and learning are organised through PBL tutorials in small groups, didactic lectures, clinical skills and laboratory practical sessions during the first three years of preclinical training. Coordinated discipline-based clerkship rotations are adopted for the next three years of clinical training. Although nutrition is usually taught in the preclinical years, opportunities for learning nutrition are very few and inadequate, as has been reported in previous studies reported from the study setting [23, 24].

### Study participants, sample size and inclusion/exclusion criteria

The study was conducted among all level 200 medical students (n = 93) who enrolled in the 2019–2020 academic year and have no nutrition background. Students with strong nutrition background such as degree in nutrition and/or have recently participated in nutrition workshop were exempted from the study.

### Intervention

The nutrition life style and behaviour change training for students (NLBCTS) was prepared by the research team members. The NLBCTS was informed by some of the recommendations reported by Lindsley et al. [25] in which they identified nutrition care competencies that medical students should develop. The NLBCTS was centred on the following thematic areas: nutrition throughout the life cycle, nutrition and health, food nutrients, nutrition assessment, malnutrition in children, nutrition and non-communicable diseases, patient counselling and motivational interviewing, nutrition in health promotion and disease prevention and referral to registered dieticians. The intervention was incorporated into an ongoing course module in which free periods were utilised. Each of the sessions lasted for 2 h. The NLBCTS was executed for a period of five weeks covering a total of 24 contact hours. The intervention was designed to supplement the increasing demand of nutrition education by medical students [22, 23], in an attempt to promote safe and effective nutrition health care practices when they graduate. Students were trained on fundamental nutrition education using multiple teaching and learning activities including interactive lecture presentations using PowerPoint, demonstrations, problem-based learning tutorials, nutrition games and role plays.

### Recruitment and data collection procedures

Students were duly informed to participate in the study by way of announcements and whatsup messages in the classrooms and whatsup group pages of the students respectively. All data was collected using a paper-based questionnaire (Supplementary material 1). Data was collected at three points i.e., at baseline, post intervention and 4-weeks follow-up assessment. In all of these instances, the students filled the questionnaire after an end of course module examination. The questionnaire contained information about the study and an informed consent to be signed before consenting to participate in the study. Voluntary participation was encouraged and students were assured of the confidentiality of their responses. During each of the measurements, students were incentivised with a sachet of yogurt upon return of a completed questionnaire.

### Data collection

All data was collected by means of a questionnaire. Student self-reported preparedness to provide nutrition care was assessed using one item derived from a previous study [22] that found the items to be valid. Using the Likert scale format, students were asked to indicate the extent to which they felt adequately prepared to provide nutrition care (i.e., 1 = very inadequate; 2 = inadequate; 3 = neither adequate nor inadequate; 4 = adequate and 5 = very adequate). Perceived relevance of nutrition education to their future practice was determined by asking students to indicate the extent to which they perceived nutrition education to be relevant to their future practice as medical doctors using a 5-point Likert scale (1 = very irrelevant; 2 = irrelevant; 3 = neither relevant nor irrelevant; 4 = relevant and 5 = very relevant). Furthermore, students need for further training in nutrition was assessed by the question: To what extent do you think you will benefit from additional nutrition education in the medical curriculum. The responses were 1 = Not at all; 2 = Slightly; 3 = moderately; 4 = considerable and 5 = significantly. Students’ assessment of the quality, appropriateness and acceptance of the intervention was assessed through an online survey tool (Google form) using a 22-item questionnaire (Refer to additional file 1). The questionnaire covered areas such as quality of the training, presentation of the didactic PowerPoint presentations, the ability of the intervention to meet its objectives and organization of the study. Using a 5-point Likert scale, students were asked to indicate their level of agreement to a list of statements. Two [2] items assessed the quality of the NLBCTS, 7 assessed the students’ perspectives regarding quality of the presentation of the intervention, 4 assessed the students’ opinion of the ability of the intervention to cover the objectives of the study and the remaining 3 questions assessed the organization and scheduling of the intervention. Five open-ended questions were included to assess the strength of the intervention, their likes and dislikes, and suggested topics for subsequent interventions and suggestions on how to improve the intervention in the future.

### Data Analysis

Data was entered into and analysed using SPSS software version 21. Descriptive statistics of mean, standard deviation and frequencies were used to describe the data. Normality test was conducted using Shapiro Wilk test to determine appropriate test techniques for data analysis. Repeated measures and Friedman tests were applied to analyse parametric and nonparametric data respectively to evaluate the difference in test score for the pre, post and 4-weeks follow up assessment. The significance level was < 0.5 at 95% confidence interval. Qualitative data from the study was analysed into themes.

### Ethical consideration

Ethical clearance for the research was obtained from the Committee on Human Research, Publications & Ethics of the Kwame Nkrumah University of Science and Technology. Written informed consent was obtained from all participants before they were enrolled into the intervention. All methods were carried out in accordance with relevant guidelines and regulations.

## Results

The mean (SD) age of the participants was 20.20 (1.70), 20.39 (1.65) and 20.30 (2.71) years at pre-intervention, post-intervention and 4-weeks follow-up respectively. Over 60% (64.5%, n = 60) of all the study participants were males. The proportion of participants who felt adequately prepared to provide nutrition care increased significantly from 38% (n = 35) to 65.2% (n = 60) immediately post-intervention and to 63.2% (n = 54) at 4-weeks follow-up (i.e., retention). The mean (SD) preparedness to provide nutrition care scores significantly (Z = 3.412, p = 0.001) increased from 3.24(0.95) at pre-intervention to 3.67 (0.81) and remained same (Z = 0.113, p = 0.910) at 4-weeks follow-up. Also, 74.2% (n = 69) of the students perceived nutrition education to be relevant to their future career as medical doctors which increased to 85% (n = 78) immediately after the intervention and declined slightly to 76% (n = 70) 4-weeks follow-up but higher than the baseline proportion. The proportion of participants who reportedly said they will benefit from further training in nutrition was 63.8% (n = 58) at pre-intervention. At post-intervention, 74.0% (n = 68) said they will benefit from further training in nutrition which decreased significantly to 52.1% (n = 48) at 4-weeks follow-up (p = 0.002).

### Students’ evaluation of the nutrition and lifestyle intervention

Students’ evaluation of the quality, appropriateness and acceptance of the intervention was conducted through an online survey tool in which 25 students responded. Among those who responded, 68% (n = 17) were male students. As shown in Table 1, majority (88%, n = 22) of the participants appraised the intervention to be of high quality. Nearly all of those who responded to the evaluation survey (96%, n = 24) felt the intervention will be beneficial to them in their future medical practice, 92% (n = 23) said they will like to participate in similar interventions in future and will recommend the intervention to others and 72% said the intervention was effective. Qualitatively, students commented that the intervention was generally effective and they were satisfied with the quality and quantity of the nutrition education provided through the intervention. This is illuminated by the following quotes from the students.

*“Lectures were effective and well understood.”* Participant 11.

*“The content was understandable and on point”.* Participant 20.

**Table 1:** Students’ evaluation of the intervention

Variable	Agree (%)	Neutral (%)	Disagree (%)
The overall quality of the training I received was high	22(88)	3(12)	0(0)
This intervention will be beneficial to me in my training as a medical doctor	24(96)	1(4)	0(0)
The methods of content delivery (Lectures, PowerPoint, etc.) were appropriate for this intervention	20(80)	5(20)	0(0)
The intervention was easy to understand and helpful	23(92)	2(8)	0(0)
The topics were presented in a logical order	20(80)	5(20)	0(0)
The vocabularies used in the workshop were clear and easy to understand	88(22)	3(12)	0(0)
Instructors where knowledgeable and effective	22(91.7)	2(8.3)	0(0)
Facilitators made use of the time allocated	18(72)	6(24)	1(4)
Facilitator(s) presentation style was effective and helpful	19(79.2)	5(20.8)	0(0)
The intervention covered the material I expected	18(72)	7(28)	0(0)
The time scheduled for the agenda items were appropriate	10(40)	13(52)	2(8)
The intervention met the training objectives	20(80)	5(20)	0(0)
The intervention met my training needs	16(64)	9(36)	0(0)
Will recommend the intervention to others	23(92)	2(8)	0(0)
Would like to have similar intervention in future	23(92)	2(8)	0(0)
Intervention has adequately prepared me for future practice	15(60)	10(40)	0(0)

### Strengths of the intervention

Students identified strengths of the intervention which were zoned into themes and quotations and presented next. They opined that the intervention provided opportunities for them to have active, interactive and practical hands-on experiences. They said the presentations provided practical demonstrations and the didactic lectures were simple and easy to understand. Aside the fact that the students identified the intervention as being very informative, they also opined that it was very interactive, entertaining, educative, simple and easy to understand.

*“It was entertaining and educative”* Participant 6.

*“It was a bit interactive, explanations were clear, delivery was good, presentations were okay…*” Participant 9.

*“It was more practical and demonstrative”* Participant 15.

Furthermore, the students reported they learned a lot of new things which they believed gave them better insights into nutrition and health and revealed to them the role of nutrition in some diseases.

*‘There was a lot to learn. I learned new things which has broadened my knowledge and also helped me with understanding medicine”*. Participant 9.

*“It enhances knowledge on the effect of nutrition on some diseases.”* Participant 14.

*“It helped me to know more about nutritional deficiencies”* Participant 16.

*“It enhanced their knowledge in various food nutrients needed in children and pregnancy”* Participant 7.

### Short comings of the intervention

The intervention had a number of challenges as identified by the students. Almost all the students lamented the timing of the lectures was challenging for them. According to most of them, the scheduled times were not convenient which made it difficult and stressful attending a number of the sessions.

*“There was no proper schedule for the intervention. Thus, coming for lectures was a bit difficult and stressful.”* Participant 14.

*“Sometimes, the duration of the lecture was long”* Participant 4.

Although the students had identified the intervention to be interactive, they identified that it was of a short duration as a result did not give them enough time to apply what they had learned.

*“The intervention was short lived.”* Participant 17.

“*There was less room to apply what has been taught or leant.”* Participant 9.

Notwithstanding the fact that the students generally felt prepared to provide nutrition care, some of them identified that the onetime intervention was not enough to make them fully prepared to provide nutrition care in their future practice. They recognised the need for the intervention to be continued throughout their training.

*“Even though the intervention was informative, the information is not adequate for my future job.*

*More interventions should be organized.* Participant 15.

*“There were fewer practical sessions, it was a bit interactive, less room to apply what has been taught or learnt.”* Participant 9.

*It should be continued in the future.”* Participant 18.

Although the students recognised that the intervention was interactive and practical some felt that they should have had more practical and interactive sessions to equip them with optimum nutritional skills that is adequate to enable them give nutrition care in their future practice.

*“The intervention was good and very helpful; I learned a lot. Thank you! I recommend that more practical sessions would be organised to help us apply what we have learnt, make understanding easier and also help you, the organisers, evaluate what we have learnt effectively.”* Participant 9.

## Discussion

In this study we evaluated the effects of a nutrition education intervention on medical students’ self-perceived preparedness to provide nutrition care, perception of the relevance of nutrition education to their future career as medical doctors and their perceived need for further training in nutrition education.

We found in this study that the percentage of students who reportedly perceived adequately prepared to provide nutrition care increased significantly from 38.0% (n = 35) at baseline to 65.2% (n = 60) immediately post-intervention and remained same at 4-weeks follow-up assessment. The observed improvement in preparedness by the study is similar to the level of preparedness documented both immediately and 2-months post-intervention by Wood et al., [26] in a culinary medicine pilot study among first-year medical students in the United States. Notwithstanding the benefits of the intervention, it is imperative to note that its one off nature may not yield the needed impact during their practice if there is no reinforcement of the gains made. There is thus the need for nutrition education to be integrated throughout the curriculum up till their final year as has been suggested by previous reports [27].

Another important finding of this study was that the proportion of students who perceived nutrition education to be relevant to their practice increased significantly from 74.2% at baseline to 84.8% post intervention. This finding is consistent with those of previous studies reporting improvement in attitudes and beliefs after following various models of nutrition education interventions [28, 29]. The improved changes in students’ self-perception of the relevance of nutrition to their future practice, shows that the intervention has impacted their attitudes and beliefs about nutrition which may result in improved nutrition practice behaviour given that attitudes and beliefs about nutrition has previously been reported to be an important determinant of both student and physician nutrition practice behaviour [28, 30].

We also found in this study that medical students’ need for nutrition education increased from 63.8% at baseline to 74.0% postintervention. Although the proportion dropped significantly lower than the baseline rate at 4-weeks post-intervention, the findings demonstrate that the one-off event of the nutrition education intervention does not meet all the needs of the medical students regards nutrition education. This is demonstrated through the results of the students’ evaluation of the intervention in which they opined that the one-off intervention is not adequate and should be integrated through the curriculum. This opinion expressed by the students is consistent with those of previous studies that have recommended the integration of nutrition education throughout the curriculum [30].

Although we find it difficult to pinpoint which aspect of the intervention was most effective in generating the improved outcomes, we will like to postulate that the effectiveness of the intervention was due to the use of interactive didactive sessions with practical demonstrations (as identified by the students’ evaluation) as well as the use of nutrition games and problem-based learning case scenarios. These innovative approaches encouraged active participation and learning. It is yet to be determined whether the gains made through this intervention will be maintained during clinical training till they graduate.

Furthermore, students’ evaluation of inadequate duration of the intervention and scheduling challenges during the course of the intervention reiterates the point that the medical curriculum is already overcrowded making it difficult to introduce stand-alone nutrition education courses or modules. There is thus the need to come out with innovative ways of integrating nutrition education into the medical curriculum. One of the ways we adopted to overcome the scheduling challenges was to integrate the sessions to be part of the timetable of an ongoing module which was a departure from our initial plan of having a standalone nutrition education intervention. This approach increased students’ participation from 40% to over 90% during the sessions. Scheduling the sessions within the module provided students with favourable times.

The strength of this study lies in our pre-post assessment design which allows us to measure the effect changes in outcomes. Another strength of this study is our reportage of students’ feedback of the intervention through the post intervention evaluation, identifying areas that future interventions could utilise to improve outcomes.

## Limitations

The study is limited by the lack of a control comparable group. This was due to the unavailability of a group with similar characteristics (i.e., same programme of study and level ) with the intervention group. The generalisability of the findings is limited given that the study was conducted in a single institution. However, the study provides insights for institutions with similar settings like ours. Furthermore, our findings are also limited by the use of self-reported measures of preparedness to provide nutrition education instead of objective measures.

## Conclusion

Our study show that an innovative, multiple-strategy nutrition education intervention can improve second year medical students’ self-perceived preparedness to provide nutrition care and their perception of the relevance of nutrition education. These are important predictors of nutrition practice behaviour and as a result an intervention of this nature assists medical schools improve the adequacy of nutrition education received by medical doctors during training. Given that the intervention has shown students’ increased need for further training in nutrition, nutrition education should be integrated throughout the curriculum to allow for reinforcement and consolidation of the gains made.

## Electronic supplementary material

Below is the link to the electronic supplementary material.

Supplementary Material 1

## Data Availability

Data is available upon request from the corresponding author.
