# Supplementary material for: Preparedness of medical students to provide nutrition care following a nutrition education intervention
Source: BMC Res Notes. 2023 May 23;16:88. doi: 10.1186/s13104-023-06348-5 (PMC10207817; doi:10.1186/s13104-023-06348-5)
Supplement: Supplementary file 1 — Supplementary Material 1 [file 13104_2023_6348_MOESM1_ESM.docx]

**Evaluation questionnaire**

**Please indicate the extent to which you agree to the following statements using 1 = Strongly disagree; 2 = Disagree; 3 = Neutral; 4 = Agree and 5 = Strongly Agree**

1. **Training Quality**
2. The overall quality of the training I received was high

1. Strongly Disagree 2. Disagree 3. Neutral 4. Agree 5. Strongly Agree

1. The intervention training will be beneficial to me in the performance of my job

1. Strongly Disagree 2. Disagree 3. Neutral 4. Agree 5. Strongly Agree

1. **Workshop Presentation**
2. The methods of content delivery (lectures, PowerPoints, etc.) were appropriate for this intervention. 1. Strongly Disagree 2. Disagree 3. Neutral 4. Agree 5. Strongly Agree
3. The intervention training was easy to understand and helpful. 1. Strongly Disagree 2. Disagree 3. Neutral 4. Agree 5. Strongly Agree
4. The topics were presented in a logical order. 1. Strongly Disagree 2. Disagree 3. Neutral 4. Agree 5. Strongly Agree
5. The vocabulary used in the workshop was clear and easy to understand. 1. Strongly Disagree 2. Disagree 3. Neutral 4. Agree 5. Strongly Agree
6. The instructors were knowledgeable and effective. 1. Strongly Disagree 2. Disagree 3. Neutral 4. Agree 5. Strongly Agree
7. The facilitators made use of the time allotted 1. Strongly Disagree 2. Disagree 3. Neutral 4. Agree 5. Strongly Agree
8. The facilitator (s) presentation style was effective in helping me learn 1. Strongly Disagree 2. Disagree 3. Neutral 4. Agree 5. Strongly Agree
9. **Workshop Objectives**
10. The intervention training covered the material I expected. 1. Strongly Disagree 2. Disagree 3. Neutral 4. Agree 5. Strongly Agree
11. The times scheduled for the agenda items were appropriate. 1. Strongly Disagree 2. Disagree 3. Neutral 4. Agree 5. Strongly Agree
12. The intervention training met the training objectives. 1. Strongly Disagree 2. Disagree 3. Neutral 4. Agree 5. Strongly Agree
13. The intervention training met my training needs. 1. Strongly Disagree 2. Disagree 3. Neutral 4. Agree 5. Strongly Agree
14. **Organisation**
15. I will recommend this intervention training to others 1. Strongly Disagree 2. Disagree 3. Neutral 4. Agree 5. Strongly Agree
16. I would you like to have similar inteventions in future

1. Strongly Disagree 2. Disagree 3. Neutral 4. Agree 5. Strongly Agree

16. The intervention has adequately prepared me for my future practice in nutrition 1. Strongly Disagree 2. Disagree 3. Neutral 4. Agree 5. Strongly Agree

What are the greatest strengths of the intervention

What did you like about the intervention training?

What did you dislike about the intervention training?

What other intervention training topics would you like to be offered by the department?

Any other comments:
